# Enhancing Interprofessional Team Performance to Prevent Medication Errors in Emergency Care: Quasi-Experimental Study Using Multimodal Virtual Simulation-Based Interprofessional Education

**DOI:** 10.2196/66999

**Published:** 2026-03-13

**Authors:** Ora-In Chu, Phanupong Phutrakool, Khrongwong Musikatavorn, Thitiporn Kongchim, Lapol Herabat, Jiraphan Ritsamdang, Krittin Bunditanukul, Kanittha Triamamornwooth, Khuansiri Narajeenron

**Affiliations:** 1 Faculty of Medicine Chulalongkorn University Bangkok Thailand; 2 Chula Data Management Center, Faculty of Medicine Chulalongkorn University Bangkok Thailand; 3 Department of Emergency Medicine, Faculty of Medicine Chulalongkorn University Bangkok Thailand; 4 Department of Emergency Medicine King Chulalongkorn Memorial Hospital, Thai Red Cross Society Bangkok Thailand; 5 Emergency Department of the Nurse Department King Chulalongkorn Memorial Hospital, Thai Red Cross Society Bangkok Thailand; 6 Department of Pharmacy Practice, Faculty of Pharmaceutical Sciences Chulalongkorn University Bangkok Thailand; 7 Clinical Pharmacy Services, Pharmacy Division King Chulalongkorn Memorial Hospital, Thai Red Cross Society Bangkok Thailand

**Keywords:** interprofessional collaboration, interprofessional education, emergency medicine, TeamSTEPPS, Team Strategies and Tools to Enhance Performance and Patient Safety, computer-based simulation, virtual simulation-based interprofessional education, medical movie, massive open online courses, co-debriefing, medication error

## Abstract

**Background:**

Effective interprofessional collaboration (IPC) is essential for patient safety; yet, poor teamwork and communication remain key challenges in high-pressure settings like the emergency department (ED), contributing to medication errors. Although Team Strategies and Tools to Enhance Performance and Patient Safety (TeamSTEPPS)–based interprofessional education addresses these issues, adaptation in clinical settings remains difficult. To bridge this gap, we developed Emergency Room Virtual Simulation-Based Interprofessional Education (ER-VIPE), a multimodal, TeamSTEPPS-integrated intervention designed to enhance IPC and reduce medication errors.

**Objective:**

The aim of the study is to evaluate the effectiveness of ER-VIPE in enhancing IPC performance among emergency physicians, nurses, and pharmacists and in reducing medication errors. The primary objective is to assess changes in IPC performance in both real-world ED settings and in computer-based simulations. The secondary objective is to examine the intervention’s impact on medication error rates in the ED.

**Methods:**

This quasi-experimental study involved 15 interprofessional teams (each comprising 1 physician, 1 pharmacist, and 2 nurses), undergoing the ER-VIPE training. This multimodal intervention included 2 medical films, a massive open online course on TeamSTEPPS and IPC, and a computer-based simulation session on acute chest pain and cardiac arrest scenarios via the simulation-based interprofessional education (SIMBIE) platform. Co-debriefings were provided as a complement to the SIMBIE session, guiding participants through positive feedback and areas of improvement. TeamSTEPPS performance was measured using the Modified TeamSTEPPS and Team Performance Observation Tool (mTPOT) in both simulation and real-world ED settings. Generalized estimating equations with a Gaussian family, identity link, and exchangeable correlation structure were used to evaluate IPC score changes. Chi-square and Fisher exact tests were applied to compare near-miss and actual medication errors before and after the intervention. A 2-tailed *P* value <.05 was considered statistically significant.

**Results:**

The study was conducted from November 2023 to January 2024 at a university hospital with 60 participants. Following the co-debriefing session in the simulation, overall mTPOT scores increased by 2.00 points (*P*<.001), with the greatest improvement among physicians (+2.70), followed by nurses (+1.75) and pharmacists (+1.56). In the ED, most mTPOT domains improved significantly across all professions 2 months after the intervention (*P*<.001). Although no significant reduction in harmful medication errors was observed, reporting of near-miss prescription errors increased significantly (*P*=.01).

**Conclusions:**

ER-VIPE enhanced IPC among ED physicians, nurses, and pharmacists, with sustained effects observed up to 2 months in real-world settings. The combination of medical films and massive open online courses provided accessible foundational knowledge, while computer-based virtual SIMBIE with co-debriefing reinforced practical communication and teamwork. Increased near-miss reporting suggests improved situational awareness and a more transparent safety culture. This multimodal training model shows promise for advancing collaboration and patient safety in emergency care.

## Introduction

Interprofessional collaboration (IPC), as defined by the World Health Organization, is the practice of multiple health workers from different professional backgrounds working together with patients, families, caregivers, and communities to deliver the highest quality of care [1]. IPC plays a critical role in improving patient care quality, safety, and outcomes by facilitating coordination in identifying needs, solving problems, and making joint decisions [2,3]. IPC is a key outcome for ensuring patient safety, and interprofessional education serves as a strategic educational approach to promote and strengthen IPC. Recognizing the global need for effective IPC, the Interprofessional Education Collaborative (IPEC) has established core competencies for interprofessional collaborative practice. These competencies are categorized into 4 major pillars: teams and teamwork, values and ethics, roles and responsibilities, and communication [4].

Despite the benefits of IPC, barriers to collaboration persist [5], contributing to medication errors, particularly medication-related harm [6]. Estimates suggest that medication errors may represent up to half of all preventable harm within health care, and these errors can be serious or even life-threatening [7]. Out of all medication error events, prescribing errors account for more than 50% of all medication errors; yet, only 30% to 70% are identified by pharmacists and nurses before administration [8-10]. Poor teamwork and interprofessional communication are major contributors to medication errors. These errors often stem from incomplete medication histories and inadequate engagement of patients and families in the care process. Ineffective team communication—such as lack of clarification, failure to verify, or neglecting to cross-check medication orders—further increases the risk, particularly during patient handoffs and in high-pressure clinical settings such as the emergency department (ED) [7,9]. The ED possesses the greatest need of IPC, where rapidly changing health care teams are required to make rapid decisions [11], often under conditions of uncertainty and resource constraints [12].

One of the most well-established frameworks for teaching IPC is Team Strategies and Tools to Enhance Performance and Patient Safety (TeamSTEPPS), developed by the US Agency for Healthcare Research and Quality (AHRQ) [13]. TeamSTEPPS provides structured training in team structure, leadership, situation monitoring, mutual support, and communication, all of which are part of the core competencies of IPC set by the IPEC. This framework is essential for enhancing collaboration and reducing errors [14]. Multiple studies have demonstrated the positive impact of TeamSTEPPS training on patient outcomes, team efficiency, and safety culture [15]. Traditional methods of TeamSTEPPS training, like e-learning and classroom-based instruction, often lack the ability to immerse learners in the high-pressure, multifactorial scenarios typical of emergency settings. Consequently, the application of TeamSTEPPS principles during hospital crises is compromised. Additionally, while TeamSTEPPS has advanced training practices, its lengthy sessions can place a heavy load on facilitators and diminish learners’ engagement.

To overcome traditional IPC TeamSTEPPS training limitations, this study introduces Emergency Room Virtual Simulation-Based Interprofessional Education (ER-VIPE) [16], a Thailand’s pioneering compact, scalable, and cost-effective virtual reality platform for IPC in emergency settings. ER-VIPE combines medical-themed movies, massive open online courses (MOOCs), and simulation-based interprofessional education (SIMBIE) with co-debriefing, and is designed to foster a no-blame culture, strengthen teamwork and communication, including patient and family engagement, and work under high-pressure conditions. The pretraining movie and MOOC components are designed to build foundational knowledge and attitudes related to the TeamSTEPPS framework, while reducing instructional burden through a self-paced, learner-directed format aligned with the flipped classroom model [17]. Following this preparatory phase, the SIMBIE component—combined with co-debriefing—offers learners the opportunity to apply these concepts in practice, grounded in experiential learning theory [18-24].

This study aims to evaluate the effectiveness of ER-VIPE in enhancing IPC performance and reducing medication errors through the application of the TeamSTEPPS framework. The primary objective is to quantitatively assess changes in IPC performance using the Modified TeamSTEPPS and Team Performance Observation Tool (mTPOT), evaluating participants both in real-world ED settings and in computer-based virtual SIMBIE sessions, following full implementation of the TeamSTEPPS-integrated ER-VIPE intervention. The secondary objective is to evaluate the intervention’s impact on medication error rates in the ED using retrospective data from hospital records collected before and after implementation of ER-VIPE among ED health care teams. The a priori hypothesis is that the implementation of ER-VIPE will improve IPC performance and will be associated with a reduction in medication error rates in the ED.

## Methods

### Research Design

Due to the rotational nature of clinical duties in the ED and logistical constraints within the real-world training environment, random assignment of participants was not feasible. Therefore, a quasi-experimental pre- and posttest design was used to evaluate the effectiveness of the multimodal TeamSTEPPS-based intervention under practical clinical conditions. This is a quasi-experimental pre- and posttest study designed to evaluate the impact of ER-VIPE integrated with the TeamSTEPPS framework as an intervention to train IPC for interprofessional health care teams and its efficacy in the reduction of medication error rates. The study is conducted in the setting of the ED at a 1500-bed teaching hospital in Thailand.

### Theoretical Framework

Each component of the ER-VIPE intervention was grounded in established educational and behavioral theories that justify its design for teaching TeamSTEPPS principles to reduce medication errors in the ED. The foundation of ER-VIPE integrates IPC with TeamSTEPPS, emphasizing team structure, communication, leadership, mutual support, and situation monitoring. These competencies are well recognized as essential for reducing prescribing, dispensing, and administration errors by strengthening shared mental models, clarifying team roles, and improving coordination among physicians, nurses, and pharmacists.

Cinemeducation and social learning theory [25] further inform the design through emotionally engaging films depicting both safe and unsafe management of high-alert drugs (HADs) and adverse drug reactions (ADRs). Observing modeled behaviors and their consequences enhances learners’ recognition of error-prone conditions and reinforces medication safety principles [26]. This foundation is complemented by experiential learning theory [27], which underpins the SIMBIE platform. SIMBIE provides deliberate, repetitive practice of medication-related teamwork behaviors within controlled scenarios, enabling learners to develop adaptive responses, strengthen role clarity, and improve communication processes essential for preventing medication errors.

Finally, the co-debriefing component is informed by social constructivism [28] and transformative learning theory [29]. Through guided reflection, dialogue, and facilitated meaning-making, learners critically examine assumptions underlying their medication practices, identify latent safety threats, and reconstruct safer mental models for IPC. This reflective process supports both individual and collective transformation, consolidating learning into long-term behavioral change [30]. Together, these interconnected frameworks provide a cohesive theoretical rationale for how ER-VIPE enhances cognitive, emotional, and team-based capacities necessary to reduce medication errors in the ED.

### Components of the ER-VIPE Training

The ER-VIPE teaching course is a multimodal virtual simulation-based interprofessional education bundle designed specifically by the research team as an innovation to teach interprofessional health care teams in team working skills to achieve the main outcome of reducing medication errors in the ED. The bundle consists of 3 major components including the medical movies, IPC course in the MOOC platform, and SIMBIE with a co-debriefing process.

#### Medical Movies

There were a total of 2 in-house production including a 48-minute movie called “Small mistakes, big consequences.” This movie covers the topic of HAD do’s and don’ts and ADR do’s and don’ts. The second movie is a 75-minute film titled “Emergency love.” Both movies were designed to demonstrate both effective and ineffective examples of teamwork in ED settings. Participants who completed the course and passed both the quiz and the final assessment had received certification prior to proceeding to the next stage of training.

Medical movies, as part of the ER-VIPE intervention, had specific learning objectives, in which participants were expected to recognize the value of IPC and TeamSTEPPS principles in improving medication safety, particularly in relation to HADs and ADRs. They were also expected to describe strategies for engaging patients and families; identify methods to prevent medication errors across prescribing, dispensing, and administration stages; and apply effective monitoring and reassessment techniques. Additionally, the course encouraged the application of metacognitive skills to help participants manage their emotions and cognitive biases during high-pressure situations.

#### Massive Open Online Courses

The MOOC platform, a publicly available, subscription-based online teaching initiative by Chulalongkorn University, aims to promote lifelong learning and public education and provides a scalable educational solution, enabling expansion of the learner base while maintaining educational quality and effectiveness through a robust online technological infrastructure. It is also a generalizable solution, applicable across diverse learner populations and educational settings [31,32].

The 1.45-hour self-paced online course on IPC for patient safety is made available through the MOOC platform. The contents were developed by contributions from experts and representative lecturers from the Faculty of Medicine, the Faculty of Nursing, the Faculty of Allied Health Sciences, the Faculty of Pharmaceutical Sciences, the Faculty of Psychology, the Faculty of Arts, the Healthcare Accreditation Institute of Thailand, and aviation training professionals.

This short course is divided into 7 soft skills modules, covering IPEC’s core competencies for IPC, TeamSTEPPS, critical team thinking for diagnosis, coping strategies for IPC, communication for IPC, 2P safety goals (patient and personnel safety), and ethical principles for IPC. The participant must complete a quiz after each module to receive a certificate of completion.

#### Computer-Based Virtual SIMBIE and Co-Debriefing Process

SIMBIE, a key component of the ER-VIPE, is an interprofessional simulation platform designed and developed by the ER-VIPE Study Group [33] in collaboration with Chulalongkorn University. It is designed using virtual simulation and 3D computer-based simulation technology [33] for virtual access through commonly available devices such as computers, enabling accessibility across institutions and adaptability to a wide range of educational contexts—thereby enhancing the platform’s generalizability. Each SIMBIE scenario is developed as a multiplayer simulation, supporting scalability for simultaneous multiuser participation. This approach ensures consistent educational quality and learner engagement, facilitated by a reliable and well-integrated online technological infrastructure [34]. The platform presents a scenario where a patient is admitted to the ED with ischemic heart disease and acute heart failure, progressing to cardiogenic shock and sudden cardiac arrest with ventricular fibrillation. Each team, consisting of a physician, a pharmacist, and 2 nurses, is responsible for managing the patient collaboratively. Players communicate via simulated telephones when avatars are located in separate areas within the game (eg, a physician in the ED and a pharmacist in the pharmacy) or through direct verbal communication when avatars are situated in the same simulated location (eg, both the physician and nurses at the patient’s bedside). Players can interact with in-game items, such as syringes and medications, by selecting them within the simulation and are expected to adhere to standard hospital protocols, including patient identification procedures using identification wristbands in the ED. A detailed description of the SIMBIE user experience has been previously reported by Narajeenron et al [16].

While managing the case, participants are evaluated by a trainer based on their respective professions, followed by a personalized co-debrief session. In this context, personalization refers to the tailored feedback and reflective discussion provided to each participant, considering their specific role, decision-making, and contributions during the scenario. This individualized approach ensures that feedback aligns with the TeamSTEPPS framework, thereby enhancing the relevance and effectiveness of the learning experience. Structured co-debriefing is conducted by 3 trained TeamSTEPPS facilitators—a physician, a nurse, and a pharmacist educator [35]. These pretrained facilitators were to observe and document key behaviors of learners during their simulation case according to the TeamSTEPPS objectives and were each assigned to lead specific TeamSTEPPS topics to provide actionable strategies for future similar situations. These strategies were coordinated by a debriefing leader to coordinate pre-debriefing, debriefing, and post-debriefing activities among the facilitators [16]. The debriefing process was delivered using the gather, analyze, summarize model [36], which promotes critical self-reflection, recognition of effective practices, and identification of areas for improvement.

The gather, analyze, summarize model supports transformative learning and fosters a growth mindset by guiding participants through 3 key phases. In the “gather” phase, participants share their experiences and emotions from the simulation, promoting self-awareness and engagement. In the “analyze” phase, facilitators lead discussions on the application of the 5 TeamSTEPPS domains in crisis situations, encouraging critical evaluation of both effective and suboptimal behaviors. The final “summarize” phase concludes with reflection on key takeaways and actionable strategies, helping participants identify behaviors to continue, modify, or discontinue in real-life situations.

After the co-debriefing, participants repeat the simulation, applying the feedback received to reinforce their learning. Trainers then re-evaluate their performance, assessing improvements and ensuring that critical concepts have been embedded into their practice.

### Participants

Participants were recruited from among 3 health care professionals working in the ED during the period of this study. Recruitment was conducted through departmental announcements and direct invitations by research assistants. Participation was voluntary and had no impact on job performance evaluations outside of this study. Informed consent was obtained from all participants prior to enrollment.

The final sample comprised 15 emergency medicine residents, 15 pharmacists (comprising 5 ED pharmacists and 10 pharmacy students on ED clerkship rotations functioning as clinical pharmacists), and 30 level 6 registered nurses (nurses who have worked in the emergency unit for over 6 years). All recruited participants are qualified to treat Emergency Severity Index level 1 and 2 patients in the ED.

### Outcome Measurements

#### Overview

**Figure 1:**
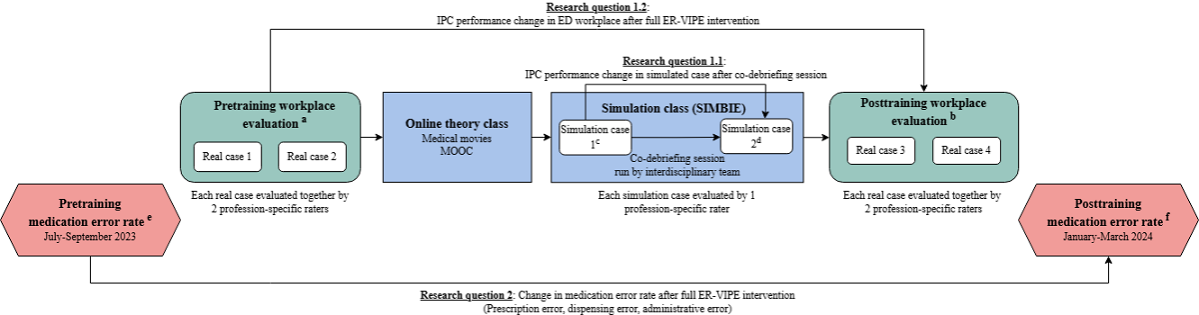
Research design and intervention overview. ED: emergency department; ER-VIPE: Emergency Room Virtual Simulation-Based Interprofessional Education; ESI: Emergency Severity Index; IPC: interprofessional collaboration; MOOC: massive open online course. aPretraining workplace IPC performance evaluations by 2 raters from the corresponding profession for 2 ESI level 1 or level 2 real cases in the ED. The assessments are conducted within 2 weeks prior to the ER-VIPE training. bPosttraining workplace IPC performance evaluations by 2 raters from the corresponding profession for 2 ESI level 1 or level 2 real cases in the ED. The assessment takes place between 10 days and 2 months after the ER-VIPE training. cPre–co-debriefing IPC performance evaluations of a simulated case by a rater (also acting as trainer) for an ESI level 2 patient with chest pain presenting with acute coronary syndrome and new-onset heart failure. dPost–co-debriefing IPC performance evaluations of a simulated case by a rater (also acting as trainer) for an ESI level 2 patient with chest pain presenting with acute coronary syndrome and new-onset heart failure. ePre–ER-VIPE intervention medication error rate in ED, recorded in a 3-month period prior to ER-VIPE training of participants. fPost–ER-VIPE intervention medication error rate in ED, recorded in a 3-month period after ER-VIPE training of participants.

Participants were assigned to undergo the ER-VIPE training in the sequence shown in Figure 1, with participants’ IPC performance based on the TeamSTEPPS framework being evaluated at 4 time points (time points a and b as workplace-based pre- and posttraining evaluation, and time points c and d as simulation-based pre- and post–co-debriefing session evaluation) using an evaluation form. Medication error rates were measured at 2 time points (time points e and f) using retrospective data.

To ensure an accurate assessment of typical workplace performance, participants were notified only 24 hours before the evaluation period. Workplace performance evaluations were scheduled according to participants’ preassigned shift schedules in the ED to minimize disruption to staffing and clinical operations.

#### Evaluation Tool for IPC Performance Based on TeamSTEPPS Framework

To assess the IPC performance of participants, we used a TeamSTEPPS and Team Performance Observation Tool (TPOT). The TPOT was published in 2004 by the AHRQ [13], in accordance with the TeamSTEPPS framework, and is an evaluation tool used to assess the quality of IPC skills according to the TeamSTEPPS framework. A Thai version of the original tool was developed and validated prior to this study [37].

Our research team had elaborated on the details of each item in the TPOT into a modified version of TPOT called mTPOT, with details of each measuring item designed to assist evaluators in assessing specific behaviors of participants, particularly those that contribute to preventing medication errors. Each item within the tool focuses on critical aspects of care, ensuring that the health care professionals perform actions that promote patient safety and reduce the risk of errors. Three sets of mTPOT were developed, each designed for physicians, nurses, and pharmacists. Each tool was specifically adapted to measure IPC behaviors expected of each professional group to support medication error reduction.

An example of the nurse evaluation within the communication domain of the TeamSTEPPS framework focuses on assessing the nurse’s ability to use the check-back technique when verifying information with the health care team, in order to ensure patient safety and accuracy during medication administration. The evaluation includes the use of patient identification techniques, requiring at least 2 of the following identifiers: patient’s name, surname, hospital number, or date of birth. Additionally, it assesses medication verification practices, including (1) correct medication name, (2) precise dosage with units (eg, 200 mg), (3) route of administration (eg, intravenous, intramuscular, and oral), (4) confirmation of diluent and volume if dilution is required, (5) frequency of administration (eg, immediately [STAT] and every 6 hours), (6) rate of administration (eg, slow intravenous push or infusion over 1 hour), and (7) duration of treatment (eg, total number of tablets or intravenous drip time).

To assess the quality of the check-back technique used by nurses, a 5-point Likert scale was used. Evaluators assigned scores based on predefined criteria reflecting the completeness and consistency of information verification. A full score of 5 was given when the nurse’s verification process is consistently performed, and complete information is received every time. A score of 4 is given when the nurse tries to verify information every time, but sometimes receives incomplete information. A score of 3 is given when verification is sometimes performed, and the nurse sometimes gets complete information. A score of 2 means that the nurse performs verification sometimes and never receives complete information each time. Finally, the lowest score of 1 is given when no verification happens when information is communicated.

The evaluation form consists of 5 sections aligned with the 5 TeamSTEPPS domains, incorporating specialty-specific behavioral indicators for each item. The team structure domain includes assessment of patient and family involvement in decision-making. The communication domain evaluates clarity, conciseness, accuracy of information exchange, use of verification techniques, and adherence to Identification, Situation, Background, Assessment, Recommendation handover protocols. The leadership domain assesses goal-setting, resource use workload distribution, and delegation. The situation monitoring domain examines the quality and frequency of patient monitoring, safety checks, and treatment follow-ups. The mutual support domain evaluates the ability to provide feedback, issue warnings, and foster a culture of teamwork.

All items in the mTPOT are scored using a 5-point Likert scale (1=very poorly done and 5=excellent, consistently done), with observers rating each participant’s behavior against specialty-specific performance standards. Higher scores reflect more frequent and proficient teamwork behaviors. Trained evaluators completed the form through direct observation, as participants managed each patient case.

An example of the TeamSTEPPS scoring tool (mTPOT) used for evaluating nurses is included in Multimedia Appendix 1 to demonstrate the types of interaction metrics assessed. Due to intellectual property restrictions, the complete set of evaluation tools for all professional groups cannot be made publicly available.

Content validation was conducted by a panel of 3 experts from each professional group to evaluate the profession-specific versions of the mTPOT. Content Validity Index (CVI) scores above 0.8 indicated strong agreement among the expert panel, confirming that the behavioral anchors demonstrated a high degree of content validity before the final version was established for use [38].

#### Medication Error Rate

##### Overview

Retrospective secondary data on medication errors, including near-miss events and miss events at all levels of ADRs in the ED, were requested from the hospital’s data center. The data were collected over a 3-month period (August to October 2023) before the ER-VIPE intervention and a 3-month period (January to March 2024) after the intervention. Medication errors were further categorized as prescription errors (PEs) by physicians, dispensing errors (DEs) by pharmacists, and administration errors (AEs) by nurses. Medication error incidents are voluntarily reported by health care team members to their supervisors or department heads for formal reporting and follow-up. Medication error data will be reported as an error rate through additional comparison with monthly ED visits and monthly prescription orders sent to the ED pharmacy.

In reporting, medication errors were categorized into 3 levels of severity: near misses that do not reach the patient, no-harm incidents that reach the patient but cause no harm, and harmful incidents that cause patient harm. Due to the voluntary nature of reporting, any increase in near-miss incidents could also correlate with a heightened detection of medication errors and a safety culture that enables medical professionals to detect errors before it reaches patients.

##### Collective and Validated Data on PE and DE

Each pharmacy unit within the ED independently collects data on PE and DE and submits these reports to the Risk Management (RM) Committee. The pharmacy department consolidates these monthly reports for validation and further analysis. The RM Committee systematically reviews the validated data to identify trends, implement preventive measures, and recommend corrective actions to mitigate the recurrence of such incidents.

##### Collective and Validated Data on AE

Each nurse in the ED independently collects incident reports on AE and submits them to the head nurse for initial review. The head nurse assesses the report, determining necessary follow-up actions, such as work review, supervisory interventions, and procedural modifications, to address the issue. Following this review, the report undergoes further validation and investigation by the supervisory inspector, who may coordinate with the pharmacy department or the patient care team as needed. The chief nursing officer then reviews and verifies the findings before submission to the RM Committee for final evaluation. The RM Committee consolidates and systematically analyzes the validated data monthly, identifying trends, implementing preventive measures, and recommending corrective actions to mitigate recurrence.

### Statistical Analysis

#### Overview

Data analysis was performed using Stata (version 15; StataCorp LLC). Each stage of the analysis was conducted according to the following methods: (1) The content validity of the mTPOT assessment form was evaluated using the CVI prior to its implementation as a performance measurement tool. (2) Participants’ demographic data (sex, age, and job experience) and baseline team performance scores, as measured by the mTPOT, were summarized using descriptive statistics. Continuous variables were reported as means with SDs and medians with IQRs, while categorical variables were presented as frequencies and percentages. (3) Baseline team performance was measured using the mTPOT. One-way ANOVA was used to compare normally distributed baseline mTPOT scores across professional groups in the initial simulated case (Multimedia Appendix 2). The Kruskal-Wallis *H* test was applied to compare nonnormally distributed workplace mTPOT scores (Multimedia Appendix 3) as well as demographic variables such as age and job experience across the 3 professions. (4) Generalized estimating equations with a Gaussian family, an identity link function, and an exchangeable correlation structure were used to measure changes in participants’ TeamSTEPPS performance (measured by mTPOT) in the ED before and after the intervention, as well as changes in TeamSTEPPS performance (measured by mTPOT) in simulation cases before and after debriefing. The average score for each domain is calculated using the total scores of each item in that section divided by the number of items assessed. The full score for each domain is 5, and the lowest score is 1. (5) Interrater reliability for each assessment was calculated using the Cohen κ coefficient, with quadratic weights to ensure the impartiality of the results. (6) Near-miss and miss events of medication errors in the ED before and after the intervention were assessed using the chi-square test and Fisher exact test. (7) A 2-tailed *P* value of <.05 was considered statistically significant for all analyses.

#### Sample Size Calculation

The sample size was determined based on a power analysis using a 2-tailed paired *t* test to compare mean differences between 2 dependent groups in a pre- and postintervention design. A total of 60 participants were required to detect an effect size of 0.37 with a significance level .05 and 80% power. This effect size, larger than a small effect (0.2) but below a medium effect (0.5), remains clinically and practically significant in this study.

### Ethical Considerations

This research was approved by the Ethical Review Committee for Research Involving Human Research Subjects, Faculty of Medicine, Chulalongkorn University (approval COA 1508/2023). All participants were informed that their participation was voluntary and that they could withdraw from the study at any time without academic or professional consequences. Written informed consent was obtained prior to data collection. Participants’ privacy and confidentiality were strictly protected; all data were anonymized and stored securely, accessible only to the research team. As compensation for their time, participants received approximately US $27 after completing the study activities. No additional incentives were provided.

## Results

### The Study Cohort

The study was conducted at a teaching hospital from November 2023 to January 2024, involving 60 interdisciplinary participants. The cohort (Table 1) comprised 15 physicians (8 first-year and 7 second-year residents), 15 pharmacists (5 ED pharmacists and 10 final-year pharmacy students rotating through the ED), and 30 ED nurses. Of the total workforce in the ED, this cohort represents 62.5% (15/24) of emergency medicine residents, 71.4% (5/7) of pharmacists in the ED pharmacy, 100% (10/10) of pharmacy students in the ED, and 29.1% (30/103) of ED nurses. The mean age of participants is 30.55 (SD 6.37) years, with 73.3% (44/60) being female and having an average experience in the ED of 6.07 (SD 7.04) years. The minimum working experience in the ED is 0 years for pharmacists, and the maximum is 28 years for nurses. Of all participants, 70% (42/60) reported no prior TeamSTEPPS training, 85% (51/60) were unfamiliar with the IPEC core competencies, and 75% (45/60) had not received any medication error prevention training.

**Table 1:** Demographic of participants by health care profession.

Factor			Overall (N=60)		Physician (n=15)		Nurse (n=30)		Pharmacist (n=15)	*P* value	
**Sex, n (%)**											.17^a^
	Female	44 (73.3)		8 (53.3)		24 (80)		12 (80)			
	Male	16 (26.7)		7 (46.7)		6 (20)		3 (20)			
**Age (years)**											
	Mean (SD)	30.55 (6.37)		27.53 (1.25)		34.27 (6.86)		26.13 (3.42)		—^b^	
	Median (IQR)	28.00 (27.00-32.50)		27.00 (27.00-28.00)		32.50 (28.00-40.00)		24.00 (23.00-29.00)		<.001^c^	
**Job experience (years)**											
	Mean (SD)	6.07 (7.04)		1.45 (1.03)		11.37 (6.46)		0.08 (0.32)		–	
	Median (IQR)	3.75 (0.42-10.00)		1.42 (0.50-2.50)		10.00 (5.42-17.00)		0.00 (0.00-0.00)		<.001^c^	
**Previous training, n (%)**											
	TeamSTEPPS^d^ (all)	18 (30)		2 (13.3)		10 (33.3)		6 (40)		.24^a^	
	TeamSTEPPS (Sim^e^)	8 (13.3)		2 (13.3)		6 (20)		0 (0)		.21^a^	
	IPEC^f^ (all)	9 (15)		2 (13.3)		2 (6.7)		5 (33.3)		.06^a^	
	IPEC (Sim)	2 (3.3)		1 (6.7)		1 (3.3)		0 (0)		>.99^a^	
	Medication error (all)	15 (25)		3 (20)		5 (16.7)		7 (46.7)		.11^a^	
	Medication error (Sim)	3 (5)		1 (6.7)		2 (6.7)		0 (0)		.80^a^	

^a^Fisher exact test.

^b^Not available.

^c^Kruskal-Wallis *H* test.

^d^TeamSTEPPS: Team Strategies and Tools to Enhance Performance and Patient Safety.

^e^Sim: simulation as a training modality.

^f^IPEC: Interprofessional Education Collaborative.

### Key Highlights

Our findings are consistent with our a priori hypothesis that the ER-VIPE intervention significantly improves IPC performance across all professional groups in both simulation case and in the ED workplace, as demonstrated by increased mTPOT scores (*P*<.001). This improvement was accompanied by an increase in the reporting of near-miss PEs (*P*=.01), likely representing clinically meaningful improvements, whereby enhanced team performance translates into safer medication practices.

### Changes in Team Performance

#### TeamSTEPPS IPC Performance Outcome in Simulated Scenarios

With 5 as the maximum score and 1 as the minimum score for each domain of TeamSTEPPS, the baseline mTPOT scores across all disciplines (Multimedia Appendix 2) averaged 2.09, with physicians scoring the lowest (1.74) and pharmacists the highest (2.49). In the mTPOT baseline scoring, the overall strength of the 15 multidisciplinary teams is team structure, while communication is the weakest area. Regarding individual specialties, physicians score lowest on mutual support (1.62), while nurses perform worst in team structure (2.00). Leadership skills are the strongest area for both physicians and nurses, scoring 1.90 and 2.13, respectively. Conversely, pharmacists scored lowest in leadership (2.24), while achieving the highest score in team structure (3.07).

**Figure 2:**
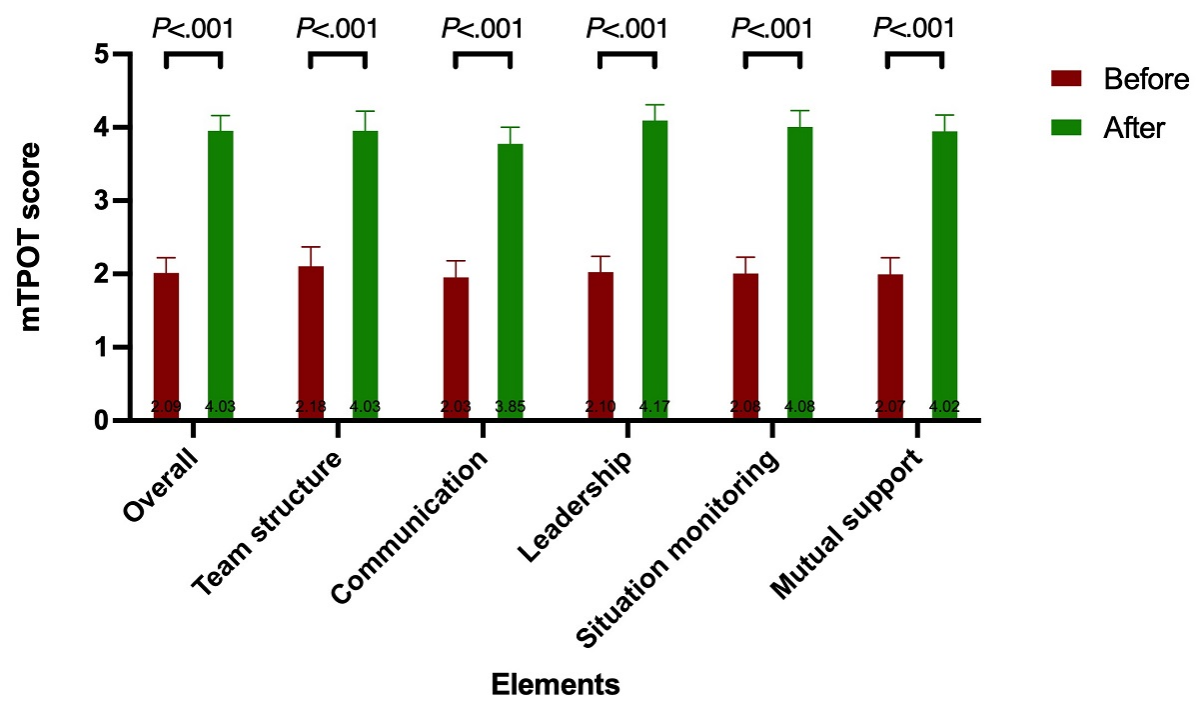
Pre- and post–co-debriefing mTPOT performance outcome in simulation scenario: overall changes across disciplines. mTPOT: Modified TeamSTEPPS and Team Performance Observation Tool.

Following the co-debriefing session after SIMBIE, the overall improvement in TeamSTEPPS performance as measured by mTPOT (Multimedia Appendix 4) was +2.00 points (*P*<.001), with physicians showing the most significant increase (+2.70) and pharmacists the least (+1.56). Nurses showed a modest increase, with an average of +1.75 points. Of the 5 domains evaluated, all showed significant improvement (*P*<.001), with leadership skills improving the most, followed by situation monitoring, mutual support, team structure, and communication (Figure 2).

**Figure 3:**
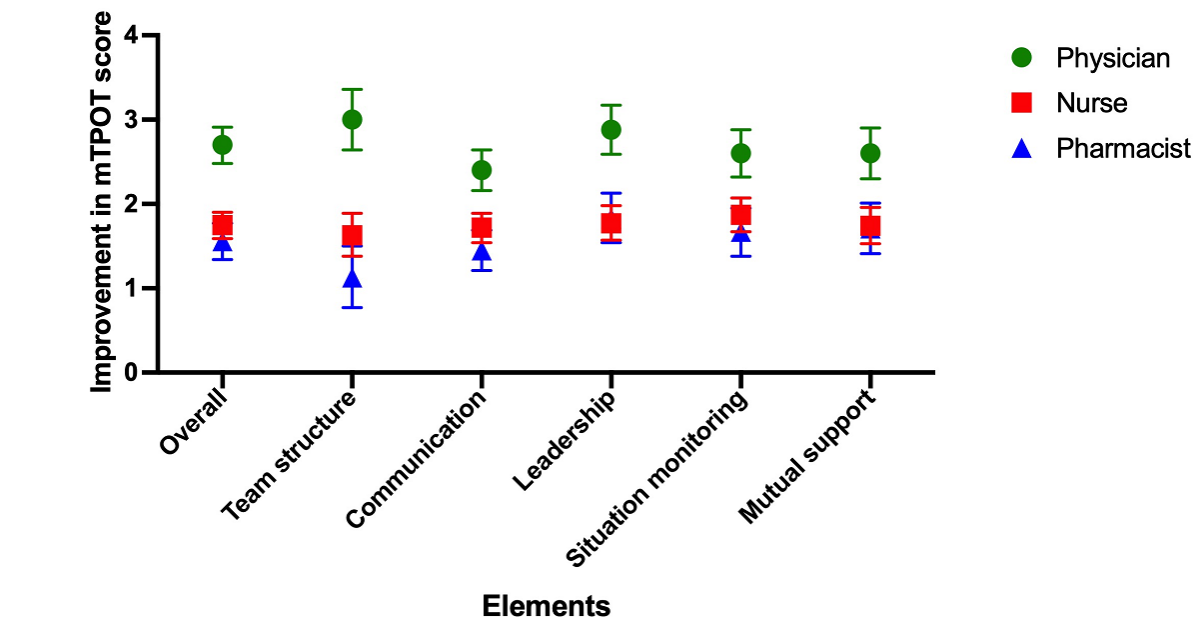
Pre- and post–co-debriefing mTPOT performance outcome in simulation scenario: breakdown by health care profession. mTPOT: Modified TeamSTEPPS and Team Performance Observation Tool.

Regarding individual specialties (Multimedia Appendix 4 and Figure 3), physicians show the greatest improvement in team structure (+3.00), followed by leadership (+2.88), a tie between situation monitoring and mutual support (+2.60), and the least improvement in communication (+2.40). Nurses showed the greatest improvement in situation monitoring (+1.87), followed by leadership (+1.77), mutual support (+1.74), communication (+1.72), and finally, team structure (+1.63). Pharmacists showed the greatest improvement in leadership, which was their weakest area during the baseline assessment (+1.83), followed by mutual support (+1.71), situation monitoring (+1.67), communication (+1.45), and the least improvement in team structure (+1.13).

#### TeamSTEPPS Team Performance Outcome in the ED Workplace

When evaluating team performance in actual emergency situations using the same mTPOT, this section aims to identify any correlation between IPC improvement in simulation and in the ED. At baseline (Multimedia Appendix 3), before any intervention, pharmacists scored lowest on the mTPOT, with a score of 2.08, while physicians achieved the highest overall score of 3.15. Prior to any intervention, all specialties score lowest in leadership (with nurses tying for the lowest score in leadership and teamwork). Among the 5 domains, physicians and pharmacists performed best in team structure, while nurses received the highest scores in situational awareness.

**Figure 4:**
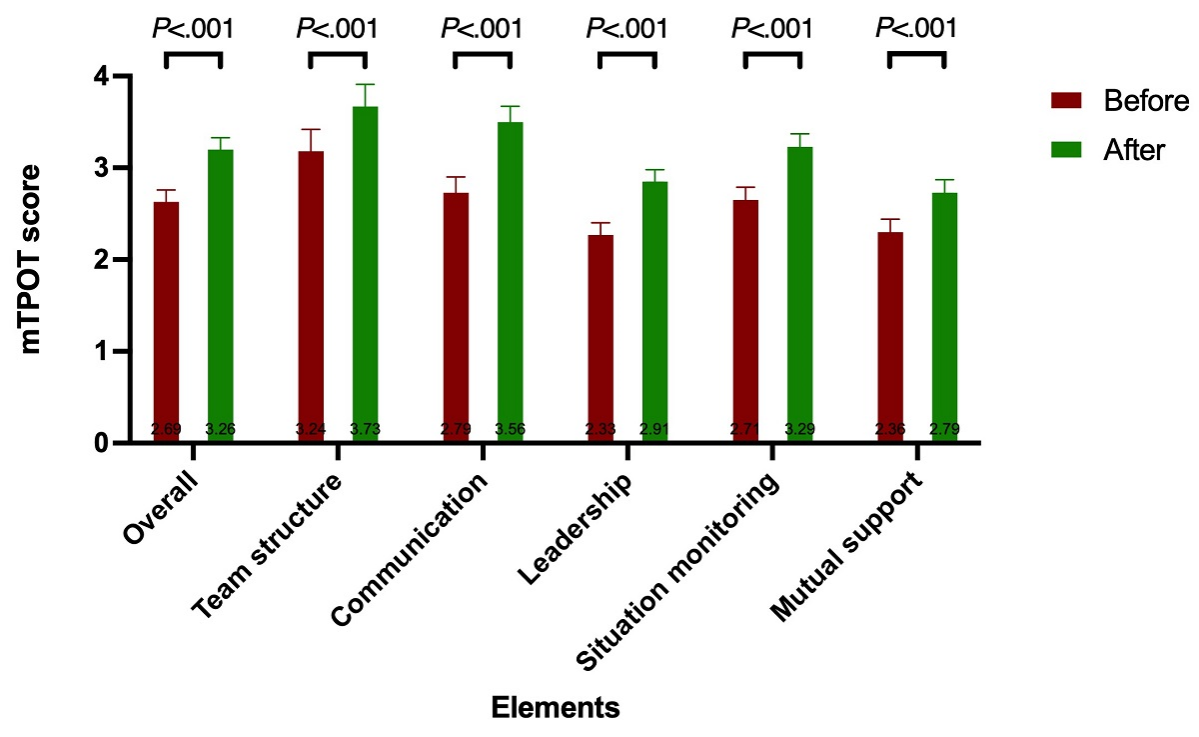
Pre- and post–ER-VIPE mTPOT performance outcome in the emergency department workplace: overall changes across disciplines. ER-VIPE: Emergency Room Virtual Simulation-Based Interprofessional Education; mTPOT: Modified TeamSTEPPS and Team Performance Observation Tool.

**Figure 5:**
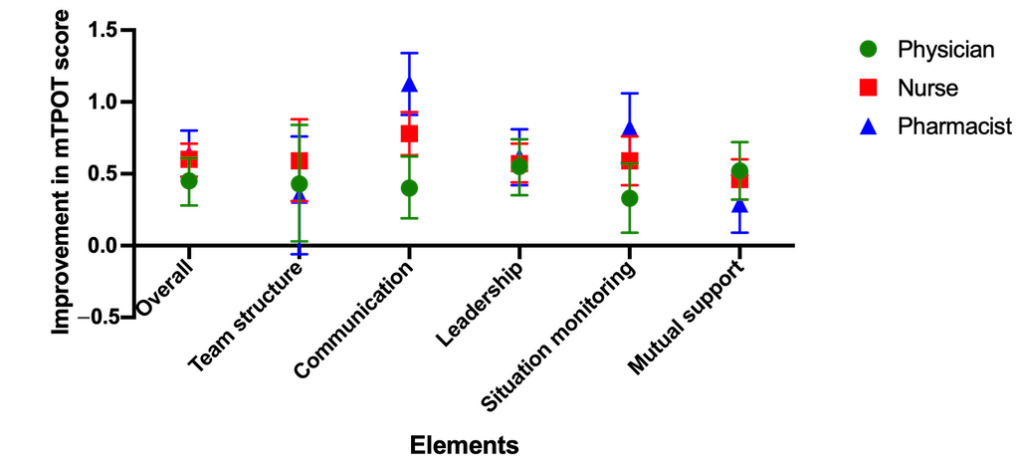
Pre- and post–ER-VIPE mTPOT performance outcome in the emergency department workplace: breakdown by health care profession. ER-VIPE: Emergency Room Virtual Simulation-Based Interprofessional Education; mTPOT: Modified TeamSTEPPS and Team Performance Observation Tool.

Overall, all specialties showed a small but significant (*P*<.001) improvement in IPC performance in the ED workplace after the intervention (Figure 4). In contrast to the baseline score (Multimedia Appendix 5), pharmacists show the greatest improvement of +0.64, while physicians show the least, with a score difference of +0.45. The individual specialties show slightly different improvement patterns (Figure 5), with leadership being the area of greatest improvement for physicians, while communication tops the list for nurses and pharmacists. Mutual support shows the least improvement for nurses and pharmacists, while situation monitoring shows the least improvement for physicians. Of the 5 domains assessed (Multimedia Appendix 5), communication skills showed the greatest improvement (+0.77), followed by leadership and situation monitoring tied (+0.58), team structure (+0.49), and mutual support (*P*<.001).

mTPOT scores in the ED were calculated based on TeamSTEPPS IPC evaluations of 2 cases by 2 independent evaluators, before and after the ER-VIPE intervention. Based on the Cohen κ coefficient [39] (Multimedia Appendix 6), each of the specialties including physician, nurse, and pharmacist demonstrates a moderate κ score of 0.53, 0.43, and 0.54, respectively, with a high percent agreement of 97.9%, 95.5%, and 87.9%, respectively.

Although the team’s performance improvement pattern in actual Emergency Severity Index level 1 or level 2 emergency cases does not mirror the simulation pattern, physicians and nurses showed significant improvement across all 5 TeamSTEPPS domains in both simulated and real-world scenarios. Pharmacists showed significant improvement across most simulation domains, with only 1 domain (team structure) showing minimal improvement.

### Changes in Miss and Near-Miss Medication Error Incidents in the ED

Retrospective data on medication errors were collected from the hospital database from August to October 2023 as preintervention baseline data and from January to March 2024 as postintervention data. Errors in each specialty were recorded separately as PE from physicians, AE from nurses, and DE from pharmacists. PE rate and AE rate were calculated in relation to the number of ED visits (preintervention: n=14,798 and postintervention: n=14,431), while the DE rate was calculated in relation to the number of prescriptions (preintervention: n=21,388 and postintervention: n=20,152; Table 2).

**Table 2:** Changes in medication error incident rate in the emergency department before (2023) and after (2024) interventions on interprofessional education.

		2023, n (%)	2024, n (%)	*P* value
**Physician (PE^a^)**		n=14,798^b^	n=14,431^c^	
	Near miss	113 (0.8)	151 (1.1)	.01^d^
	No harm incident	0 (0)	0 (0)	—^e^
	Harmful incident	0 (0)	1 (0)	.49^f^
**Nurse (AE^g^)**		n=14,798^b^	n=14,431^c^	
	Near miss	4 (0)	3 (0)	>.99^f^
	No harm incident	2 (0)	8 (0.1)	.06^f^
	Harmful incident	1 (0)	0 (0)	>.99^f^
**Pharmacist (DE^h^)**		n=21,388^i^	n=20,152^j^	
	Near miss	4 (0)	3 (0)	>.99^f^
	No harm incident	9 (0)	9 (0)	>.99^f^
	Harmful incident	0 (0)	0 (0)	—

^a^PE: prescription error.

^b^Number of emergency department (ED) visits (during August-October 2023).

^c^Number of ED visits (during January-March 2023).

^d^Chi-square test.

^e^Not available.

^f^Fisher exact test.

^g^AE: administration error.

^h^DE: dispensing error.

^i^Number of prescription orders from the ED to ED pharmacists (during August-October 2023).

^j^Number of prescription orders from the ED to ED pharmacists (during January-March 2023).

Without significant changes in staffing or ED policies, the number of near-miss incidents due to PE increased significantly from 0.8% to 1.1% (*P*=.01). This finding indicates an increase in the reporting of near-miss PEs that were identified and intercepted by the health care team before reaching patients. There is no significant difference in other categories of medication errors across other specialties (Table 2).

## Discussion

### Principal Findings

This study demonstrates a significant improvement in TeamSTEPPS performance, as measured by mTPOT scores, in both simulated and real-world ED settings. However, despite a high percentage agreement, the moderate interrater reliability observed in the real-world ED setting warrants cautious interpretation, as variability in rater judgment may have affected score consistency. Among the 5 TeamSTEPPS domains measured in the ED setting, communication showed the most substantial improvement, followed by situation monitoring and leadership. While evaluating IPC performance over a 10- to 63-day period after ER-VIPE, a consistent enhancement in teamwork behaviors was recorded by mTPOT, alongside an increase in near-miss prescribing errors, indicating improved error detection and reporting.

### In-Depth Evaluation by Profession

#### Physician: Enhanced Leadership Skills

Effective communication with colleagues and strong leadership skills are crucial aspects of professionalism for emergency physicians [40]. For physicians, the mTPOT scores reflected a marked improvement in the leadership domain in the TeamSTEPPS framework. A key scoring criterion in this domain measures the physician’s ability to consult or notify other professions, such as the pharmacist in the use of HAD for high-risk patients to reduce medication errors, and was able to delegate tasks to others, such as pharmacists for medication preparation, medication reconciliation, allergy history collection, drug interaction monitoring, and dose adjustments based on patient factors such as weight, liver function, and kidney function. These skills were particularly beneficial in minimizing medication-related risk in high-risk patient groups, including children, pregnant women, and older patients [41].

Another scoring criterion in the leadership domain measures the use of “brief, huddle, and debrief” in team huddles. Physicians, often as leaders of the health care team, engaged in discussions with other health care professionals, ensuring that the entire team had a shared understanding of the situation, goals, and management plan. This ongoing communication helps maintain team alignment and enable real-time adaptive decision-making throughout the service process. This action also enables health care teams to more efficiently and effectively detect potential medication errors before they occur [42].

#### Nurses and Pharmacists: Strengthened Communication

Workplace-based evaluation of both nurses and pharmacists shows significantly improved mTPOT scores in communication skills. In ER-VIPE, TeamSTEPPS communication skills including closed-loop communication, use of verification techniques, handover techniques, and the correct use of information sources were taught to participants. The improved mTPOT scores in the workplace thus reflect that these skills were effectively enhanced in the workplace after the intervention, where pharmacists and nurses were able to communicate more effectively, with greater clarity, conciseness, and accuracy. In the team setting, the mTPOT scores also reflect that while performed minimally prior to the ER-VIPE intervention, nurses and pharmacists showed an increased ability to help the health care team in gathering information from various sources, including direct communication with patients and family members, and double-checking medication information through an external data source. The strengthened communication skills do not only lead to increased efficiency and timeliness in the completion of emergency tasks [43] but also allow the interprofessional team to support each other in gathering patients’ health information, such as drug history, and acting synergistically in cross-checking on the correct use of medications to prevent medication errors [44].

#### Situation Monitoring Impact on Clinical Practice and Patient Safety

For nurses and pharmacists, situation monitoring is the second most improved domain in the workplace. This improvement, along with enhanced communication, has been shown to contribute to better clinical outcomes by preventing medication errors and identifying patient safety threats [45]. An example of this can be seen in the mTPOT evaluation rubrics where nurses demonstrated increased awareness of changes in patients’ condition before administering medication.

After the ER-VIPE intervention, mTPOT scores of the nurses reflect an increased awareness of events where colleagues might be at risk of making unsafe decisions, such as preparing to administer medication without performing an independent double-check using the “7 Rights” of medication administration (right patient, right drug, right dose, right route, right time, right reason, and right documentation) [46]. This shared situational awareness enabled team members to intervene before the occurrence of medication errors, enhancing the overall safety of medication administration processes [47].

Shifting away from a culture of underreporting—driven by cognitive biases, frustration with limited changes, or fear of embarrassment and professional repercussions [7,48], the increase in reported near-miss PEs following ER-VIPE training reflects a growing awareness among health care staff, particularly in nurses and pharmacists, of the importance of shared situational awareness through near-miss medication error reporting. By maintaining a blame-free culture for a safe medication error reporting environment, the transparency in near-miss data can further help authorities in identifying systemic issues and address root causes of errors, leading to overall improvements in patient safety [49].

Finally, the practice of using “thinking aloud” for shared situational awareness in the TeamSTEPPS framework allows the interprofessional team to create a more cohesive understanding of the patient’s condition and care strategies. By ensuring all team members were “on the same page,” the teams were better equipped to prevent medication errors due to incomplete history such as drug allergies, identification of strengths and weaknesses in clinical decisions, and other potential risks, contributing to safer and more efficient patient care [50].

### Implications: Enhancing ED Pharmacists’ Skills and Fostering a Culture of IPC for Medication Error Reduction Through ER-VIPE

This study presents an innovative multimodal virtual simulation-based interprofessional training approach in emergency settings with TeamSTEPPS principles. It further enhances our understanding of how virtual simulation, particularly in emergency settings, plays a crucial role in developing the skills of ED pharmacists. Previous studies by Bunditanukul et al [51] have highlighted the effectiveness of simulation in fostering positive teamwork attitudes among ED pharmacists. Our findings build on this, showing that training not only improves attitudes but also significantly enhances pharmacists’ performance in both simulated and real-world ED settings.

In the simulated environment, the pharmacist subgroup showed a modest improvement. We anticipated that this was a result of the limited potential magnitude of change with a relatively high baseline mTPOT score. Despite the smaller margin of improvement, their final scores remained higher than those of the nurse subgroup (Multimedia Appendix 4). Conversely, in the real-world ED observations, pharmacists demonstrated a lower baseline team performance, which provided greater room for growth. In this setting, the pharmacist group achieved a marked improvement, showing the largest gain among all professional groups (Multimedia Appendix 5).

We speculate that the higher baseline mTPOT scores in the simulation compared with the real-world workplace setting may be explained by 2 factors. First, the existing siloed work culture within the ED may have contributed to a fixed professional mindset, limiting IPC in daily practice. Second, participants had completed the medical movie and MOOC modules prior to the baseline simulation assessment. These preparatory learning activities likely served as a supportive primer, fostering a more positive mindset toward teamwork and communication, thereby elevating their baseline performance during the simulation.

Taken together, the consistently high posttraining mTPOT scores across both simulation and real-world settings highlight the strong responsiveness of pharmacists to the ER-VIPE training. In the current clinical context—where the role of clinical pharmacists in the ED remains limited—these results underscore ER-VIPE’s potential to strengthen pharmacists’ competencies and support their integration into interprofessional emergency care teams.

The role of ED pharmacists is an emerging field that can greatly benefit from enhanced IPC training. This study serves as a model for expanding TeamSTEPPS IPC training applications, particularly in training clinical pharmacists to support ED physicians and core nursing teams. Emerging ED pharmacists are expected to play a key role in identifying and addressing medication discrepancies that may currently be overlooked [52,53]. ER-VIPE can be used to train them to work confidently alongside other health care professionals in the ED [16], especially since pharmacists often report a lack of experience or familiarity in the clinical setting [54].

Given the dynamic nature of clinical cases and team dynamics in emergency care, effective TeamSTEPPS IPC training should involve ongoing practice and peer feedback. This approach not only enhances individual skills but also fosters a culture of teamwork and ongoing improvement among the team. By implementing regular feedback mechanisms, we can ensure that the trained multiprofessional ED members, including pharmacists, are well-equipped to handle evolving challenges and contribute to improving patient outcomes.

### Comparison With Prior Work

Compared to previous research, there is evidence supporting the link between educational interventions and improved patient outcomes, as shown in the literature. However, these studies typically used conventional simulation training methods rather than virtual approaches. For instance, studies conducted in China by Shi et al [14] have shown a positive impact of patient safety education programs and their return on investment. However, these studies did not leverage the scalability and generalizability offered by virtual simulation, which is a distinct advantage of the ER-VIPE intervention [15].

This study is one of the first to track behavioral changes in the ED setting following IPC training, rather than confining observations to classroom simulations alone. We designed the data collection process to include evaluation of multiple patient interactions per assessment point, with each expert evaluator observing more than 1 clinical encounter per study session, enhancing the robustness of our findings. Another factor that enhanced this reliability was our team’s development of a tailored mTPOT tool for the Thai language context, customized to meet the needs of the respective professional groups. This tool underwent comprehensive validation, achieving a CVI above 0.8 prior to its implementation in our study.

These results not only validate the effectiveness of the ER-VIPE training as a scalable and cost-effective model for continuous professional development in health care [15] but also demonstrate its applicability as a general educational tool for improving workplace performance in the ED (Kirkpatrick Evaluation Level 3). Furthermore, these findings highlight the importance of IPC in enhancing patient safety outcomes in real-world clinical settings (Kirkpatrick Evaluation Level 4) [55,56]. This is a step further from the previous study where TeamSTEPPS training had been shown to improve team members’ perception and attitudes about teamwork (Kirkpatrick Evaluation Level 2-3) [57].

The ER-VIPE training aligns with the latest TeamSTEPPS 3.0 model, launched by AHRQ in 2024, which introduces key updates such as greater patient involvement in medical decisions and enhanced active learning strategies, including discussions, online group exercises, and video-based simulations [58]. ER-VIPE builds on this new version by incorporating a local context—for example, fostering a no-blame culture in co-debriefing sessions to address the root causes of medication errors. Additionally, with local trainers, we tailored the training to cultural nuances, such as tone of voice and precise word choice, to enhance participant engagement and understanding.

### Limitations

Several limitations should be acknowledged. First, variations in emergency patient cases—such as severity, urgency, and staffing—may have influenced team dynamics and communication, potentially affecting mTPOT scores regardless of the intervention.

Although a high percentage of agreement, the moderate reliability suggests that while the mTPOT provides a structured framework for assessment, future refinements could enhance consistency. Strategies such as enhanced rater training, standardized scoring protocols, or double-checking ratings with additional raters could be considered to improve agreement.

The study design also limits generalizability. The absence of randomization and a control group introduces potential self-selection bias and restricts the ability to attribute improvements solely to the intervention. Additionally, the voluntary nature of participation may have led to self-selection bias, as more motivated individuals were likely to enroll and perform better.

Due to the nature of voluntary reporting, this study could not rule out the risk of underreporting. However, to the best of our knowledge, we had observed a significant increase in reporting in the workplace of near-miss PEs, which we are hopeful that our effort has helped minimize potential harmful medication errors in the ED. In addition, the cohort of our study consisted of 62.5% (15/24) emergency medicine residents, 71.4% (5/7) ED pharmacists, and only 29.1% (30/103) ED nurses. The smaller proportion of nurses who underwent the ER-VIPE intervention may have contributed to fewer reported incidents of medication errors in both AE and DE categories, which, in real-world practice, are often identified through nursing surveillance and reporting.

Finally, the potential influence of the Hawthorne effect should be considered. Although participants may have altered their behavior due to being observed, this effect still reflects improved awareness and understanding of appropriate teamwork practices—aligning with the principle of “fake it until you make it” in the development of professional behaviors.

### Future Studies

Future research should use more rigorous designs, such as randomized controlled trials or matched control groups, to strengthen causal inferences. Longitudinal studies with repeated measures and blinding could also help minimize the Hawthorne effect and better assess sustained behavioral change and its impact on medication error reduction.

Further investigation is needed to determine the optimal frequency of retraining to maintain IPC skills and to reinforce a culture of safety and open communication. Expanding the sample size and tracking missed or near-miss events over time would provide a clearer picture of long-term impact and sustainability.

Although this study did not demonstrate a significant reduction in harmful medication errors, it revealed meaningful improvement in IPC performance, even with limited participation among ED professionals, particularly nurses (30/103, 29.1%). This finding underscores the need for future studies to increase the proportion of nurse participants and to incorporate targeted training aimed at enhancing nurses’ ability to detect and report adverse and near-miss medication events. Collectively, these directions could guide the development of scalable, evidence-based IPC and TeamSTEPPS programs to strengthen teamwork, cross-disciplinary communication, and patient safety in emergency care.

### Conclusions

The multimodal virtual SIMBIE tool (ER-VIPE), integrated with TeamSTEPPS, effectively improved IPC among ED residents, nurses, and pharmacists, with sustained impact up to 2 months. The increase in near-miss medication error reporting suggests heightened awareness and a stronger safety culture. This approach shows promise in promoting team communication, error reporting, and developing dedicated ED teams to address medication errors at their root causes.

## Appendix

Multimedia Appendix 1 Modified Team Performance Observation Tool for nurses.

Multimedia Appendix 2 Baseline simulated scenario Modified Team Performance Observation Tool score in each health care discipline.

Multimedia Appendix 3 Baseline emergency department workplace case Modified Team Performance Observation Tool score in each health care discipline.

Multimedia Appendix 4 Simulated scenario Modified Team Performance Observation Tool score change in each health care discipline after co-debriefing session.

Multimedia Appendix 5 Emergency department workplace case Modified Team Performance Observation Tool score change in each health care discipline after Emergency Room Virtual Simulation-Based Interprofessional Education intervention.

Multimedia Appendix 6 Interrater reliability by specialty.

## Abbreviations

ADR: adverse drug reaction
AE: administration error
AHRQ: Agency for Healthcare Research and Quality
CVI: Content Validity Index
DE: dispensing error
ED: emergency department
ER-VIPE: Emergency Room Virtual Simulation-Based Interprofessional Education
HAD: high-alert drug
IPC: interprofessional collaboration
IPEC: Interprofessional Education Collaborative
MOOC: massive open online course
mTPOT: Modified TeamSTEPPS and Team Performance Observation Tool
PE: prescription error
RM: Risk Management
SIMBIE: simulation-based interprofessional education
TeamSTEPPS: Team Strategies and Tools to Enhance Performance and Patient Safety
TPOT: Team Performance Observation Tool
