# Supplementary material for: Enhancing Interprofessional Team Performance to Prevent Medication Errors in Emergency Care: Quasi-Experimental Study Using Multimodal Virtual Simulation-Based Interprofessional Education
Source: JMIR Med Educ. 2026 Mar 13;12:e66999. doi: 10.2196/66999 (PMC13032089; doi:10.2196/66999)
Supplement: Multimedia Appendix 1 [file mededu_v12i1e66999_app1.pdf]

Modified Team Performance Observation Tool (mTPOT) for Nurses - Translation version

v1.2_01.11.2023

Overall Scoring criteria

(5) Excellent, perfect, done properly every time.

(4) Well done, mostly done.

(3) Should have done it more often/more regularly. But it is acceptable.

(2) Did not do well/did poorly, should have done more often.

(1) Did very poorly/should have done but did not do.

---

1. Team structure

1.4 Let patients and relatives be part of the team.

Observe teamwork in which patients and relatives are included as part of the team, for example:

1.Take a patient's history such as drug allergies and previous drug use from patients or relatives before giving medication to patients.

2. Give the patient and/or relatives (in case the patient is unconscious) the opportunity to clarify concerns or ask questions.

3. Involve the patient or relative (in case the patient is unconscious) in deciding to consent before giving medication, especially high-risk medicine (inform & verbal consent)

4. Giving advice on medication use. Watching for side effects and/or symptoms that require returning to the hospital

- ☐ (5) Excellent, perfect, done properly every time.
- ☐ (4) Well done, mostly done.
- ☐ (3) Should have done it more often/more regularly. But it is acceptable.
- ☐ (2) Did not do well/did poorly, should have done more often.
- ☐ (1) Did very poorly/should have done but did not do.

---

2. Communication2.1 Forward information between team members in a clear, concise, accurate, and timely manner.

Observed behavior:

Talking in a clear, concise, and correct manner means communicating information about medication at the right time.
1.1 Communicate clearly.
1.2 Communicate concisely.
1.3 Convey accurate and comprehensive information.

- ☐ (5) Excellent, perfect, done properly every time.
- ☐ (4) Well done, mostly done.
- ☐ (3) Should have done it more often/more regularly. But it is acceptable.
- ☐ (2) Did not do well/did poorly, should have done more often.
- ☐ (1) Did very poorly/should have done but did not do.

2.2 Search for information from all available sources.

Observe the completeness of information gathered from every available source, such as

from the patient
from relatives
Review EMR such as history of drug allergies, use of previous medications
Search for information from reliable sources such as NICE guidance, EBM guidelines, comprehensive drug information database and clinical decision support such as Lexicomp, Poison Centers.
Call a pharmacist or a medicine expert to consult.

- ☐ (5) Excellent, perfect, done properly every time.
- ☐ (4) Well done, mostly done.
- ☐ (3) Should have done it more often/more regularly. But it is acceptable.
- ☐ (2) Did not do well/did poorly, should have done more often.
- ☐ (1) Did very poorly/should have done but did not do.

2.3 Use verification techniques. (check-backs) to check communicated information.

Observed behavior:

There is verification every time information is communicated, especially.
1. Identify patient (using 2/3 patient identifiers: Name and surname, HN, and DOB)
2.1 Medication name
2.2 Dosage with units (e.g., 200 mg)
2.3 Route of Administration (e.g., IV, IM, oral)
2.4 Dilution details (i.e., diluent AND volume) if applicable
2.5 Frequency (e.g., stat, q6h)
2.6 Rate (e.g., slowly push, drip in 1 hr.) if applicable.
2.7 Duration (e.g., 90 tabs, iv drip over 6 hours)

Note: The duration may be determined by the number of pills and the frequency of use if it is an oral medication.

- ☐ (5) Verify information every time it is communicated and receive a complete information every time.
- ☐ (4) Verify information every time it is communicated but still received incomplete information each time.
- ☐ (3) Verification is sometimes performed where information is communicated and sometimes gets complete information.
- ☐ (2) Verification is sometimes performed where information is communicated and never received complete information each time.
- ☐ (1) There is no verification when information is communicated.

2.4 Use ISBAR, and handover techniques for effective communication between team members.

Observed behavior:

ISBAR is used in each communication especially in the case of medicine.
1. I is from Being able to introduce yourself and the patient ID at least 2/3 of the total (full name/ HN/ date of birth).
2. S is from Situation: to inform the situation
3. B is from Background: to tell relevant clinical history.
4. A is from Assessment: Evaluate and predict what the problem is.
5. R is from Recommendation and Request: What do you want to do or what help do you need?

- ☐ (5) Can complete all 5 steps every time (100%)
- ☐ (4) Can complete all 5 steps and complete them almost every time (at least 80%).
- ☐ (3) Can complete all 5 steps, but completes them only sometimes (at least 50%), and there are times when all 5 steps are not completed.
- ☐ (2) Can complete all 5 steps, but does not do them every time (less than 50%), and there are times when all 5 steps are not completed.
- ☐ (1) Did not follow all 5 steps at all.

---

3. Leadership3.1 Identify the team's goals and vision.

Observed behavior

A Brief, Huddle or Debrief is clearly communicated to the team from the beginning to report problems and diagnosis and treatment plans (especially in targeted drug-related issues).

- ☐ (5) Brief, Huddle or Debrief, communicate issues perfectly and appropriately every time from the start (within 5 minutes)
- ☐ (4) Brief, Huddle or Debrief report problems very well, mostly within 10 minutes of starting the case.
- ☐ (3) Brief, Huddle or Debrief report problems inconsistently but acceptable, occurring more than 10 minutes since the start of the case.
- ☐ (2) Brief, Huddle or Debrief reports problems poorly, unclearly or incompletely and/or more than 15 minutes from the start of the case.
- ☐ (1) There is no Brief, Huddle or Debrief to report problems and diagnosis and maintenance plans to the team.

3.2 Use resources efficiently to achieve maximum team performance.

Observed behaviors such as

Able to seek advice from a doctor or pharmacist at the time of need and at an appropriate time.
Seek assistance from your fellow nurse when needed. (Manage time resources and prioritize work well)
Prepare equipment and tools correctly and appropriately for the medication that will be given to the patient, such as preparing an infusion pump, drug administration set, premix, syringe size.

- ☐ (5) Excellent, perfect, done properly every time.
- ☐ (4) Well done, mostly done.
- ☐ (3) Should have done it more often/more regularly. But it is acceptable.
- ☐ (2) Did not do well/did poorly, should have done more often.
- ☐ (1) Did very poorly/should have done but did not do.

3.3 Maintain an appropriate balance of workload in the team.

Observed behavior:

Nurses observe the workload of assigned teammates and provide feedback or divide/distribute work appropriately to reduce medication errors caused by human factors.

- ☐ (5) Excellent, perfect, done properly every time.
- ☐ (4) Well done, mostly done.
- ☐ (3) Should have done it more often/more regularly but it is acceptable.
- ☐ (2) Did not do well/did poorly, should have done more often.
- ☐ (1) Did very poorly/should have done but did not do.

3.4 Assign work or things to do as considered appropriate.

Observed behaviors such as:

Able to divide work correctly and appropriately to Airway Nurse and Circulating Nurse to reduce medication errors caused by human factors (for example, doctors and nurses should not give the medication to patients alone, especially without independent double check).

- ☐ (5) Excellent, perfect, done properly every time.
- ☐ (4) Well done, mostly done.
- ☐ (3) Should have done it more often/more regularly. But it is acceptable.
- ☐ (2) Did not do well/did poorly, should have done more often.
- ☐ (1) Did very poorly/should have done but did not do.

3.5 There are brief, huddles, and debriefs.

Observed behavior (Especially problems related to medication):

- Briefing is performed.
- Huddle is performed.
- Debrief is performed.

- ☐ (5) There was a brief, huddle and debrief, and there was clear and useful communication (3/3)
- ☐ (4) There was a brief, huddle, and debrief, but there were still some communication flaws (3/3)
- ☐ (3) Missing one of these: brief, huddle, and debrief (2/3)
- ☐ (2) Missing two of these: brief, huddle, and debrief (1/3)
- ☐ (1) No brief, huddle and debrief at all (0/3)

3.6 Be a role model for good teamwork behavior.

Observed behavior:

- Respect your co-workers, use a polite tone, and always end a sentence with polite particles (krub/ka)
- Willing to accept objections and criticisms from team members.
- Say “thank you” to each other. (Promote a supporting culture in in working environment)

*Create an atmosphere and culture of communication and teamwork for medication safety.

- ☐ (5) Nurses can perform all 3 items excellently.
- ☐ (4) Nurses can perform all 3 items, but is not yet complete.

- ☐ (3) Nurses can perform only 2/3 of the questions.
- ☐ (2) Nurses can perform only 1/3 of the questions or 2/3 of the questions, but still need to improve, such as tone of voice, use of polite particles, etc.
- ☐ (1) Nurses did not perform all 3 items (0/3) or performed them poorly. Need to improve a lot.

---

4. Situation monitoring

4.1 Patient symptoms are monitored.

Observed behavior:

Nurses keep monitoring vital signs and evaluate the patient's condition periodically.
1. Before prescribing medication
2. During medication
3. After the patient received medication
4. Before transferring the patient to another department or other personnel, or before discharge

- ☐ (5) There is periodic and complete monitoring of patient symptoms (4/4 items).
- ☐ (4) There is periodic monitoring of patient symptoms, only 3/4 items.
- ☐ (3) There is periodic monitoring of patient symptoms, only 2/4 items.
- ☐ (2) There is only 1/4 periodic monitoring of patient symptoms.
- ☐ (1) There is no monitoring of the patient's condition at all (0/4 items)

4.2 Check teammates for safety and preventing errors.

Observed behavior:

Detected,helped monitor, or noticedthat other members of the team, such as fellow doctors, nurses, and pharmacists, were about to make an unsafe mistake, such as:
1. Giving medicine for unreasonable, dangerous, or prohibited reasons. (Inappropriate or Irrational Use of Medicine)
2. Did not ask for patient consent or giving patients the opportunity to refuse medication. (Right to refuse)
3. Gave medication without independent double check 7R during medicine preparation before giving it to the patient.
4. Gave medication without identifying the patient, such as not asking for their full name norlooking at the patient's wrist tag. (Wrong patient)
5. Forgot to complete documentation about medicines, such as patient drug allergies, prescriptions, and progress notes.

- ☐ (5) Excellent, perfect, done properly every time.
- ☐ (4) Well done, mostly done.
- ☐ (3) Should have done it more often/more regularly. But it is acceptable.
- ☐ (2) Did not do well/did poorly, should have done more often.
- ☐ (1) Did very poorly/should have done but did not do.

4.4 Review result of treatment and identify changes that may result in adjustments to the care plan.

Observed behavior:

- The work of team members is periodically followed up to ensure that it aligns with the plans and goals set. Ensure safety of patient and team through face-to-face conversation by checking and following up.
- Give patients and relatives an opportunity to ask questions, provide additional information and participate in treatment planning. (Informed consent)
- Check the performance of pharmacists and/or doctors in helping with drug allergy history and medical reconciliation verbally or by reading the EMR.
- Check that the patient has received the medication according to the treatment plan.
- Evaluate, observe symptoms and vital signs of patients after receiving medication.

- ☐ (5) The work of team members is followed up completely. Five items are completed (5/5)
- ☐ (4) The work of team members is followed up, but not completely. Four items are completed (4/5)
- ☐ (3) The work of team members is followed up, but not completely. Three items are completed (3/5)
- ☐ (2) The work of team members is followed up, but not completely. Two items are completed (2/5)
- ☐ (1) There is no follow-up on the work of team members at all or did very poorly (0-1/5)

4.5 There is communication to ensure that everyone in the team sees the same problem, situation, and solution.

Observed behavior:

The SA that has been acknowledged is shared through communicating (Think Aloud) verbally or recorded in the EMR so that the relevant teams are on the same page by understanding the problems, various solutions, and preventative measures, such as

1. Problem of emergency in ABC resuscitation
2. Problems with the chief complaint (Problem lists)
3. Possible diseases (Differential diagnosis) which may be related to the medication that the patient uses.
4. Drug allergy history

5. Risk of drug resistance history
6. Risk of prescribing medicine to the wrong person (Identification error)
7. Risk of prescribing the wrong medicine (LASA, Look Alike Sound Alike)

☐ (5) Excellent, perfect, done properly every time.
☐ (4) Well done, mostly done.
☐ (3) Should have done it more often/more regularly. But it is acceptable.
☐ (2) Did not do well/did poorly, should have done more often.
☐ (1) Did very poorly/should have done but did not do.

---

5. Supporting each other

5.1 Provide support for each other's work.

Observed behavior:

1. Help each other take a history of drug allergies from patients or relatives
2. Help take medical reconciliation history from the patient or relatives.
3. Help monitor patients' symptoms and vital signs.
4. Help give advice about medicine to patients and relatives/caregivers.

☐ (5) Nurses are able to help each other in all 4 items (4/4)
☐ (4) Nurses are able to help each other in 3 items (3/4)
☐ (3 Nurses are able to help each other in 2 items (2/4)
☐ (2) Nurses are able to help each other in 1 item (1/4)
☐ (1) Nurses did not perform all 4 items (0/4)

5.2 Provide constructive and appropriate suggestions to team members.

Observed behavior

Give constructive feedback at the right time (constructive feedback) such as recommending the use of prefill syringe MO, premix levophed or saying thank you when team members give suggestions on drug use, drug interactions or drug allergies across groups, etc.

☐ (5) Give constructive feedback in a timely manner and say “thank you” appropriately whenever team members help in a completely accurate manner, both in quantity and quality.
☐ (4) Give appropriate feedback and say “thank you” when team members help but still have areas for improvement or are not performing as well as they should. (Good but not perfect for both quantity and quality)

- ☐ (3) Give feedback and say “thank you” when team members help but are not performing those enough or have areas that need improvement, such as feedback that is not constructive or not timely. (Should do this more often/more regularly, but it is acceptable.)
- ☐ (2) Give feedback and say “thank you” when team members help but perform too little and have areas that need improvement, such as feedback that is not constructive or not timely. (Should be improved greatly in quantity and quality)
- ☐ (1) There is no constructive feedback or thank you to team members at all.

5.3 The principle of warning is used without causing distress. Double warning or use of CUS principles to ensure patient safety

Observed behavior:

There are words of objection and warning in a non-distressing way using CUS, for example:
1. Problem of emergency in ABC resuscitation
2. Possible diseases (Differential diagnosis) which may be related to the medicines the patient uses.
3. Risk of drug allergy
4. Risk of drug resistance
5. Risk of giving medicine to the wrong person (Identification error)
6. Risk of giving the wrong medicine (LASA, Look Alike Sound Alike)
7. Warning in cases where communication does not receive a call back or does not comply with ISBAR principles, etc.

- ☐ (5) Excellent, perfect, done properly every time.
- ☐ (4) Well done, mostly done.
- ☐ (3) Should have done it more often/more regularly. But it is acceptable.
- ☐ (2) Did not do well/did poorly, should have done more often.
- ☐ (1) Did very poorly/should have done but did not do.

5.4 The Double Warning Rule, or DESC, is used to resolve conflicts.

Observed behaviors in the ER:

There is a second warning if the team member does not hear or has not yet taken action.
DESC is used to resolve conflicts (if any).
*Especially problems related to medication

- ☐ (5) There is a second warning if team members do not hear or no action has been taken every time, and DESC is used if there is a conflict (2/2) and is used completely and correctly.

- ☐ (4) There is a second warning if team members do not hear or no action has been taken every time, and DESC is used if there is a conflict (2/2), but it is not used completely.
- ☐ (3) There is a second warning if the team members do not hear or have not yet taken action every time, or DESC is used if there is a conflict (1/2) and is used completely and correctly.
- ☐ (2) There is a second warning if team members do not hear or action has not yet been taken every time, or DESC is used if there is a conflict (1/2) but it is not used completely.
- ☐ (1) There is no second warning if the team member does not hear or has not yet taken action and DESC is not used.
- ☐ N/A
