# Supplementary material for: Enhancing Interprofessional Team Performance to Prevent Medication Errors in Emergency Care: Quasi-Experimental Study Using Multimodal Virtual Simulation-Based Interprofessional Education
Source: JMIR Med Educ. 2026 Mar 13;12:e66999. doi: 10.2196/66999 (PMC13032089; doi:10.2196/66999)
Supplement: Multimedia Appendix 2 [file mededu_v12i1e66999_app2.docx]

| **TeamSTEPPS Score** | **Overall**  **(n=60)** | **Physician**  **(n=15)** | **Nurse**  **(n=30)** | **Pharmacist (n=15)** | **P value** |
| --- | --- | --- | --- | --- | --- |
| **Overall** |  |  |  |  |  |
| Mean±SD | 2.09±0.62 | 1.74±0.27 | 2.07±0.59 | 2.49±0.74 | .003 |
| Median (IQR) | 1.98 (0.86) | 1.63 (0.42) | 1.93 (0.89) | 2.53 (1.18) | - |
| **Team Structure** |  |  |  |  |  |
| Mean±SD | 2.18±1.00 | 1.67±0.72 | 2.00±0.79 | 3.07±1.10 | - |
| Median (IQR) | 2.00 (2.00) | 2.00 (1.00) | 2.00 (1.00) | 3.00 (2.00) | .001 |
| **Communication** |  |  |  |  |  |
| Mean±SD | 2.03±0.64 | 1.70±0.27 | 2.07±0.66 | 2.30±0.75 | - |
| Median (IQR) | 1.88 (1.00) | 1.75 (0.50) | 2.00 (0.75) | 2.25 (1.25) | .06 |
| **Leadership** |  |  |  |  |  |
| Mean±SD | 2.10±0.62 | 1.90±0.35 | 2.13±0.63 | 2.24±0.78 | - |
| Median (IQR) | 2.00 (0.83) | 1.83 (0.67) | 2.08 (1.00) | 2.33 (1.33) | .51 |
| **Situation Monitoring** |  |  |  |  |  |
| Mean±SD | 2.08±0.66 | 1.83±0.54 | 2.05±0.67 | 2.38±0.69 | .07 |
| Median (IQR) | 2.00 (1.00) | 1.75 (0.75) | 2.00 (0.75) | 2.25 (1.25) | - |
| **Mutual Support** |  |  |  |  |  |
| Mean±SD | 2.07±0.71 | 1.62±0.40 | 2.09±0.63 | 2.47±0.86 | .003 |
| Median (IQR) | 2.00 (0.83) | 1.67 (0.67) | 2.17 (0.66) | 2.67 (1.33) | - |
| One-way ANOVA | | | | | |
