# Supplementary material for: Enhancing Interprofessional Team Performance to Prevent Medication Errors in Emergency Care: Quasi-Experimental Study Using Multimodal Virtual Simulation-Based Interprofessional Education
Source: JMIR Med Educ. 2026 Mar 13;12:e66999. doi: 10.2196/66999 (PMC13032089; doi:10.2196/66999)
Supplement: Multimedia Appendix 3 [file mededu_v12i1e66999_app3.docx]

| **mTPOT Score** | **Overall**  **(n=60)** | **Physician**  **(n=15)** | **Nurse**  **(n=30)** | **Pharmacist (n=15)** | **P value** |
| --- | --- | --- | --- | --- | --- |
| **Overall** |  |  |  |  |  |
| Mean±SD | 2.68±0.46 | 3.14±0.25 | 2.76±0.18 | 2.06±0.34 | - |
| Median (IQR) | 2.74 (0.53) | 3.11 (0.37) | 2.76 (0.20) | 2.04 (0.50) | <.001 |
| **Team Structure** |  |  |  |  |  |
| Mean±SD | 3.23±0.75 | 3.38±0.44 | 2.78±0.26 | 3.95±1.03 | - |
| Median (IQR) | 3 (0.75) | 3.5 (0.75) | 2.75 (0.25) | 4.5 (1.25) | <.001 |
| **Communication** |  |  |  |  |  |
| Mean±SD | 2.78±0.53 | 3.24±0.26 | 2.87±0.31 | 2.12±0.48 | - |
| Median (IQR) | 2.94 (0.63) | 3.25 (0.31) | 2.94 (0.38) | 2.06 (0.75) | <.001 |
| **Leadership** |  |  |  |  |  |
| Mean±SD | 2.33±0.66 | 2.92±0.25 | 2.54±0.23 | 1.32±0.37 | - |
| Median (IQR) | 2.52 (0.77) | 2.92 (0.33) | 2.54 (0.33) | 1.17 (0.75) | <.001 |
| **Situation Monitoring** |  |  |  |  |  |
| Mean±SD | 2.71±0.76 | 3.15±0.28 | 3.07±0.20 | 1.55±0.63 | - |
| Median (IQR) | 3 (0.53) | 3.06 (0.44) | 3.13 (0.31) | 1.38 (0.88) | <.001 |
| **Mutual Support** |  |  |  |  |  |
| Mean±SD | 2.36±0.67 | 3.01±0.29 | 2.54±0.19 | 1.36±0.39 | - |
| Median (IQR) | 2.54 (0.71) | 3 (0.42) | 2.58 (0.25) | 1.17 (0.67) | <.001 |
| Kruskal Wallis H test | | | | | |
