# Supplementary material for: Enhancing Interprofessional Team Performance to Prevent Medication Errors in Emergency Care: Quasi-Experimental Study Using Multimodal Virtual Simulation-Based Interprofessional Education
Source: JMIR Med Educ. 2026 Mar 13;12:e66999. doi: 10.2196/66999 (PMC13032089; doi:10.2196/66999)
Supplement: Multimedia Appendix 4 [file mededu_v12i1e66999_app4.docx]

| **Factor** | **Before**  **(95% CI)** | **After**  **(95% CI)** | **Difference**  **(95% CI)** | **P value** |
| --- | --- | --- | --- | --- |
| **Overall** | 2.09 (1.96, 2.22) | 4.03 (3.90, 4.16) | 2.00 (1.89, 2.11) | <.001 |
| Physician | 1.74 (1.48, 2.01) | 4.44 (4.18, 4.70) | 2.70 (2.48, 2.91) | <.001 |
| Nurse | 2.07 (1.88, 2.25) | 3.81 (3.63, 4.00) | 1.75 (1.59, 1.90) | <.001 |
| Pharmacist | 2.49 (2.23, 2.75) | 4.05 (3.79, 4.31) | 1.56 (1.34, 1.77) | <.001 |
| **Team Structure** | 2.18 (2.00, 2.37) | 4.03 (3.85, 4.22) | 1.92 (1.73, 2.11) | <.001 |
| Physician | 1.67 (1.30, 2.03) | 4.67 (4.30, 5.03) | 3.00 (2.64, 3.36) | <.001 |
| Nurse | 2.00 (1.74, 2.26) | 3.63 (3.37, 3.89) | 1.63 (1.38, 1.89) | <.001 |
| Pharmacist | 3.07 (2.70, 3.43) | 4.20 (3.83, 4.57) | 1.13 (0.77, 1.50) | <.001 |
| **Communication** | 2.03 (1.89, 2.18) | 3.85 (3.71, 4.00) | 1.86 (1.73, 1.98) | <.001 |
| Physician | 1.70 (1.41, 1.99) | 4.10 (3.81, 4.39) | 2.40 (2.16, 2.64) | <.001 |
| Nurse | 2.07 (1.86, 2.27) | 3.78 (3.58, 3.99) | 1.72 (1.54, 1.89) | <.001 |
| Pharmacist | 2.30 (2.01, 2.59) | 3.75 (3.46, 4.04) | 1.45 (1.21, 1.69) | <.001 |
| **Leadership** | 2.10 (1.96, 2.24) | 4.17 (4.02, 4.31) | 2.16 (2.01, 2.32) | <.001 |
| Physician | 1.90 (1.62, 2.18) | 4.78 (4.49, 5.06) | 2.88 (2.59, 3.17) | <.001 |
| Nurse | 2.13 (1.93, 2.33) | 3.91 (3.71, 4.11) | 1.77 (1.57, 1.98) | <.001 |
| Pharmacist | 2.24 (1.96, 2.53) | 4.08 (3.79, 4.36) | 1.83 (1.54, 2.13) | <.001 |
| **Situation Monitoring** | 2.08 (1.93, 2.23) | 4.08 (3.93, 4.23) | 2.04 (1.89, 2.19) | <.001 |
| Physician | 1.83 (1.54, 2.13) | 4.43 (4.14, 4.73) | 2.60 (2.32, 2.88) | <.001 |
| Nurse | 2.05 (1.84, 2.26) | 3.92 (3.71, 4.12) | 1.87 (1.67, 2.07) | <.001 |
| Pharmacist | 2.38 (2.09, 2.68) | 4.05 (3.76, 4.34) | 1.67 (1.38, 1.95) | <.001 |
| **Mutual Support** | 2.07 (1.91, 2.22) | 4.02 (3.86, 4.17) | 2.02 (1.86, 2.18) | <.001 |
| Physician | 1.62 (1.31, 1.93) | 4.22 (3.91, 4.53) | 2.60 (2.30, 2.90) | <.001 |
| Nurse | 2.09 (1.87, 2.31) | 3.83 (3.61, 4.05) | 1.74 (1.53, 1.96) | <.001 |
| Pharmacist | 2.47 (2.16, 2.78) | 4.18 (3.87, 4.49) | 1.71 (1.41, 2.01) | <.001 |
| Generalized Estimating Equations (GEE) | | | | |
