# Supplementary material for: Enhancing Interprofessional Team Performance to Prevent Medication Errors in Emergency Care: Quasi-Experimental Study Using Multimodal Virtual Simulation-Based Interprofessional Education
Source: JMIR Med Educ. 2026 Mar 13;12:e66999. doi: 10.2196/66999 (PMC13032089; doi:10.2196/66999)
Supplement: Multimedia Appendix 5 [file mededu_v12i1e66999_app5.docx]

| **Factor** | **Before**  **(95% CI)** | **After**  **(95% CI)** | **Difference**  **(95% CI)** | **P value** |
| --- | --- | --- | --- | --- |
| **Overall** | 2.69 (2.61, 2.76) | 3.26 (3.18, 3.33) | 0.57 (0.49, 0.86) | <.001 |
| Medicine | 3.15 (3.02, 3.28) | 3.60 (3.47, 3.73) | 0.45 (0.28, 0.61) | <.001 |
| Nurse | 2.76 (2.67, 2.85) | 3.36 (3.27, 3.45) | 0.60 (0.48, 0.71) | <.001 |
| Pharmacist | 2.08 (1.95, 2.20) | 2.72 (2.59, 2.84) | 0.64 (0.48, 0.80) | <.001 |
| **Team Structure** | 3.24 (3.05, 3.42) | 3.73 (3.55, 3.91) | 0.49 (0.29, 0.69) | <.001 |
| Medicine | 3.41 (3.09, 3.73) | 3.84 (3.52, 4.16) | 0.43 (0.03, 0.84) | .04 |
| Nurse | 2.78 (2.55, 3.01) | 3.38 (3.15, 3.60) | 0.59 (0.31, 0.88) | <.001 |
| Pharmacist | 3.98 (3.66, 4.29) | 4.33 (4.01, 4.64) | 0.35 (-0.06, 0.76) | .09 |
| **Communication** | 2.79 (2.69, 2.90) | 3.56 (3.46, 3.67) | 0.77 (0.66, 0.88) | <.001 |
| Medicine | 3.27 (3.10, 3.44) | 3.67 (3.50, 3.85) | 0.40 (0.19, 0.62) | <.001 |
| Nurse | 2.87 (2.75, 3.00) | 3.65 (3.52, 3.78) | 0.78 (0.63, 0.93) | <.001 |
| Pharmacist | 2.15 (1.98, 2.32) | 3.27 (3.10, 3.45) | 1.13 (0.91, 1.34) | <.001 |
| **Leadership** | 2.33 (2.26, 2.40) | 2.91 (2.84, 2.98) | 0.58 (0.48, 0.68) | <.001 |
| Medicine | 2.92 (2.78, 3.05) | 3.46 (3.33, 3.60) | 0.55 (0.35, 0.74) | <.001 |
| Nurse | 2.54 (2.45, 2.64) | 3.12 (3.02, 3.21) | 0.57 (0.44, 0.71) | <.001 |
| Pharmacist | 1.33 (1.19, 1.46) | 1.94 (1.80, 2.08) | 0.62 (0.42, 0.81) | <.001 |
| **Situation Monitoring** | 2.71 (2.63, 2.79) | 3.29 (3.21, 3.37) | 0.58 (0.46, 0.70) | <.001 |
| Medicine | 3.15 (2.98, 3.31) | 3.48 (3.31, 3.64) | 0.33 (0.09, 0.57) | .006 |
| Nurse | 3.07 (2.95, 3.19) | 3.66 (3.54, 3.78) | 0.59 (0.42, 0.76) | <.001 |
| Pharmacist | 1.55 (1.39, 1.72) | 2.38 (2.21, 2.54) | 0.82 (0.58, 1.06) | <.001 |
| **Mutual Support** | 2.36 (2.29, 2.44) | 2.79 (2.72, 2.87) | 0.43 (0.33, 0.53) | <.001 |
| Medicine | 3.01 (2.86, 3.15) | 3.52 (3.38, 3.67) | 0.52 (0.32, 0.72) | <.001 |
| Nurse | 2.54 (2.44, 2.65) | 3.00 (2.90, 3.10) | 0.46 (0.32, 0.60) | <.001 |
| Pharmacist | 1.36 (1.21, 1.50) | 1.64 (1.50, 1.79) | 0.29 (0.09, 0.49) | .001 |
| Generalized Estimating Equations (GEE) | | | | |
