# Supplementary material for: Enhancing Interprofessional Team Performance to Prevent Medication Errors in Emergency Care: Quasi-Experimental Study Using Multimodal Virtual Simulation-Based Interprofessional Education
Source: JMIR Med Educ. 2026 Mar 13;12:e66999. doi: 10.2196/66999 (PMC13032089; doi:10.2196/66999)
Supplement: Multimedia Appendix 6 [file mededu_v12i1e66999_app6.docx]

| **Factor** | **Weighted Kappa (kw)** | | |
| --- | --- | --- | --- |
|  | **Percent Agreement** | **Kappa** | **Interpretation** |
| Physician | 97.92% | .53 | Moderate |
| Nurse | 95.51% | .43 | Moderate |
| Pharmacist | 87.94% | .54 | Moderate |
